# SUV_max_ reduction predicts long-term survival in patients of non-pCR both in the tumor and lymph nodes after neoadjuvant chemoradiotherapy in esophageal squamous cell carcinoma

**DOI:** 10.1186/s12957-021-02208-3

**Published:** 2021-04-09

**Authors:** Yushi Nagaki, Satoru Motoyama, Yusuke Sato, Akiyuki Wakita, Hiromu Fujita, Yoshihiro Sasaki, Kazuhiro Imai, Yoshihiro Minamiya

**Affiliations:** 1 Division of Esophageal Surgery, Akita University Hospital, Akita, Japan; 2 Department of Thoracic Surgery, Akita University Graduate School of Medicine, 1-1-1 Hondo, Akita, 010-8543 Japan

**Keywords:** ESCC, NACRT, non-pCR, SUVmax

## Abstract

**Background:**

A pathological complete response (pCR) after neoadjuvant chemoradiotherapy (NACRT) ensures long-term survival in esophageal squamous cell carcinoma (ESCC) patients following esophagectomy, but pCR patients are a minority. The aim here was to identify prognostic factors in patients with non-pCR ESCC after NACRT.

**Methods:**

This is a retrospective study. Investigated were 5-year overall survival (OS), disease-specific survival (DSS), and relapse-free survival (RFS) among non-pCR ESCC patients divided into pT0N0, primary site pCR (pT0N+), lymph node pCR (pT+N0), and non-pCR in both the tumor and lymph node (pT+N+) subgroups after NACRT and esophagectomy. Focusing on the SUV_max_ reduction rate in the primary tumor in 88 patients who underwent FDG-PET before and after NACRT, we used univariate and multivariate Cox proportional hazard models to identify prognostic factors.

**Results:**

Although there were no significant survival differences among non-pCR ESCC patients with pT0N+, pT+N0, or pT+N+, survival rate among pT+N+patients was the poorest. After setting a 60% cutoff for the SUV_max_ reduction rate in the tumor, RFS curves for non-pCR patients significantly differed between patients above the cutoff and those below it. For pT+N+ patients, the SUV_max_ reduction rate (<60% vs ≥ 60%) was an independent prognostic factor of OS, DSS, and RFS.

**Conclusion:**

Because ESCC patients with SUV_max_ reduction rates of <60% in the tumor after NACRT and categorized as pT+N+ after NACRT had significantly poorer prognoses, even after esophagectomy, a change in treatment strategy may be an option to improve survival.

## Background

Esophageal cancer, particularly at advanced stages, is a highly aggressive tumor with a poor prognosis. Esophageal squamous cell carcinoma (ESCC) accounts for approximately 90% of all esophageal cancer cases worldwide and is the most common histological subtype in Asia, Africa, and South America [1]. Because many ESCC patients are diagnosed with locally advanced tumors with lymph node metastasis, multimodal treatment combining surgery with chemotherapy and/or radiotherapy has been introduced to improve patient prognosis [1]. In this effort, several clinical trials have shown the efficacy of neoadjuvant chemotherapy (NACT) and neoadjuvant chemoradiotherapy (NACRT) [2–9]. NACRT leads to marked down-staging of ESCC tumors, and a pathological complete response (pCR) to NACRT is a key factor contributing to survival in patients with esophageal cancer [10]. On the other hand, little has been reported about the prognosis of non-pCR patients, who account for the majority ESCC patients treated with NACRT. Specifically, few studies have focused on the difference in prognosis between primary tumor pCR (pT0N+), lymph node pCR (pT+N0), and non-pCR of both primary tumor and lymph node (pT+N+) in patients with ESCC.

^18^F-fluorodeoxyglucose-positron emission tomography (FDG-PET) is a functional imaging examination that has been used to assist in the diagnosis of cancer, particularly diagnosis of metastasis and recurrence. The maximum standardized uptake value (SUV_max_), a parameter of FDG-PET, is a measure of the highest FDG uptake within a region of interest. It has been reported that the SUV_max_ reduction rate in the primary tumor after NACRT is a valuable predictor of pathological therapeutic effect and the survival of patients of esophageal cancer [11]. In the present study, we used relative changes in the SUV_max_ after NACRT to investigate the key factors contributing to long-term survival in non-pCR patients.

## Methods

### Patients

Included in this retrospective study were 115 consecutive patients with confirmed thoracic ESCC with lymph node metastasis and without distant metastasis. But patients with supraclavicular lymph node metastasis were included because supraclavicular lymph nodes were considered to be locoregional node metastasis in Japan. The patients were treated with NACRT followed by esophagectomy at Akita University Hospital between 2009 and 2017. NACRT was recommended for patients with either a clinical T3-4 primary tumor or regional lymph node metastasis, and with an Eastern Cooperative Oncology Group performance status (ECOG PS) of 0–1. Clinical staging was determined according to the TNM classification of the UICC (8th edition) [12] based on upper gastrointestinal endoscopy, esophagography, contrast-enhanced CT, and FDG-PET.

### Neoadjuvant chemoradiotherapy (NACRT)

The NACRT protocol entailed radiotherapy (40.0–41.4 Gy in 20–23 fractions) with two courses of combined chemotherapy composed of 5-fluorouracil (5-FU) 800 mg/m^2^/day on days 1–5 plus cisplatin (FP) or nedaplatin (FGP) 80 mg/m^2^/day on day 1. High-energy X-rays (10 MV) were used for the radiotherapy. All patients underwent 3-dimensional radiotherapy planning, and the radiotherapy target was set around the gross tumor volume and metastatic lymph nodes.

### ^18^F-fluorodeoxyglucose-positron emission tomography (FDG-PET)

FDG-PET was standard to perform before NACRT and then 3–4 weeks after NACRT, before surgery. However, since it was performed only on outpatients, FDG-PET was not performed on hospitalized patients with severe esophageal stenosis due to esophageal cancer. The FDG-PET was performed with one scanner, and procedure was standardized by radiologist. Patients fasted for at least 4 h before receiving an intravenous injection of 185 MBq/kg FDG (FDGscan; Nihon Medi-Physics) and then rested for 1 h before scanning. All images were acquired using a combined PET/CT scanner (Discovery ST Elite 16; GE Healthcare). Low-dose CT images were acquired using a 16-detector row scanner with the following parameters: 120 kV, auto mA (noise index 25.0), 0.6 s tube rotation, 3.75-mm section thickness, 512×512 matrix, and 70-cm field of view. The CT scan was acquired while the patient engaged in shallow breathing and covered an area from the upper thigh to the base of the skull. The SUV_max_ was established for each patient by drawing regions of interest around the primary tumor on attenuation-corrected FDG-PET images and then calculating the value using software integrated into the PET/CT scanner. The formula used for the calculation was as follows: SUV_max_ = (*C* [mCi/ml]/ID[mCi])/body weight, where *C* was the activity at a pixel within the tissue identified as a region of interest, and ID was the injected dose per kilogram of body weight. The SUV_max_ reduction rate was calculated as follows: {(baseline SUV_max_) − (SUV_max_ after NACRT)}/(baseline SUV_max_) × 100%.

### Surgery

Esophagectomy was scheduled to be performed more than 3 weeks after completing NACRT, by which time patients had no treatment-related adverse events worse than grade 2 according to the Common Terminology Criteria for Adverse Events (CTCAE) Version 4.0 [13]. Esophagectomy under right thoracotomy or thoracoscopic esophagectomy with extended 3-field lymph node dissection was performed.

### Pathological analysis

The surgically resected thoracic ESCC specimens were subjected to routine pathological examination. We divided the patients into pCR and non-pCR groups based on the pathological diagnosis. Additionally, non-pCR patients were subdivided into pT0N+, pT+N0, or pT+N+.

### Statistical analysis

Continuous variables are presented as the median (range: minimum–maximum). Differences between groups were analyzed using the chi-square test to compare non-continuous variables and the Kruskal-Wallis test for continuous variables. Overall survival (OS), disease-specific survival (DSS), and relapse-free survival (RFS) curves were estimated using the Kaplan-Meier method and were compared using the log-rank test. The univariate Cox proportional hazard model was used to assess associations between clinicopathological characteristics and survival. Multivariate analysis was used to adjust for the effects of other variables. All statistical analyses were performed using JMP14 (SAS Institute). All the *P* values were reported as two-sided with a significance level of 0.05.

## Results

### Patients and tumor characteristics

Of 115 ESCC patients enrolled in this retrospective study, chemotherapy consisting of FP was used for 43 (37%) patients, while FGP was used for 73 (63%) patients. The interval between NACRT and surgery was 40 (21–95) days. Surgery entailed complete esophageal resection (R0) in all patients. The median number of dissected lymph nodes was 49 (8–97). We categorized 25 (22%) patients as pT0N0, 10 (8%) as pT0N+, 40 (34%) as pT+N0, and 40 (34%) as pT+N+. Their characteristics are shown in Table 1. The median length of follow-up for censored cases was 55.5 (18–120) months after surgery. Forty-six (40%) patients experienced recurrences during the observation period. Of those, 37 (80%) recurrences were distant metastasis, and 9 (20%) were local metastasis.

**Table 1:** Clinicopathological features of the ESCC patients enrolled in this retrospective study

Characteristic, ***n*** (%)	pCR	non-pCR			***P***
	pT0N0	pT0N1-3	pT1-4N0	pT1-4N1-3	
	***n***=25	***n***=10	***n***=40	***n***=40	
**Median age (range)**	63 (44–77)	65 (53–75)	66 (43–74)	63 (41–75)	*0.2402*
**Gender**					0.7178
**Female**	5 (20.0)	1 (10.0)	8 (20.0)	5 (12.5)	
**Male**	20 (80.0)	9 (90.0)	32 (80.0)	35 (87.5)	
**Tumor location**					0.8228
**Upper**	5 (20.0)	2 (20.0)	8 (20.0)	9 (22.5)	
**Middle**	9 (36.0)	4 (40.0)	18 (45.0)	21 (52.5)	
**Lower**	11 (44.0)	4 (40.0)	14 (35.0)	10 (25.0)	
**Tumor differentiation**					0.5425
**Not poorly**	20 (80.0)	8 (80.0)	36 (90.0)	36 (90.0)	
**Poorly**	5 (20.0)	2 (20.0)	4 (10.0)	4 (10.0)	
**Depth of invasion (cT)**					0.0567
**T1-2**	5 (20.0)	3 (30.0)	2 (5.0)	3 (7.5)	
**T3-4**	20 (80.0)	7 (70.0)	38 (95.0)	37 (92.5)	
**Lymph node metastasis (cN)**					0.5858
**N1**	18 (72.0)	8 (80.0)	26 (65.0)	24 (60.0)	
**N2-3**	7 (28.0)	2 (20.0)	14 (35.0)	16 (40.0)	
**Distant metastasis (cM)**					0.2138
**M0**	19 (76.0)	10 (100)	36 (90.0)	35 (87.5)	
**M1 (lymph node)**	6 (24.0)	0 (0)	4 (10.0)	5 (12.5)	
**Clinical stage(cStage)**					0.1051
**I**	1 (4.0)	1 (10.0)	2 (5.0)	0 (0)	
**II**	3 (12.0)	2 (20.0)	0 (0)	3 (7.5)	
**III**	14 (56.0)	6 (60.0)	34 (85.0)	31 (77.5)	
**IVA**	1 (4.0)	1 (10.0)	0 (0)	1 (2.5)	
**IVB**	6 (24.0)	0 (0)	4 (10.0)	5 (12.5)	
**Recurrence**					0.0456^a^
**Presence**	5 (20.0)	4 (40.0)	15 (37.5)	22 (55.0)	
**Absence**	20 (80.0)	6 (60.0)	25 (62.5)	18 (45.0)	
**Prognosis**					0.3115
**Alive**	22 (88.0)	6 (60.0)	27 (67.5)	24 (60.0)	
**Dead with ESCC**	2 (8.0)	3 (30.0)	10 (25.0)	14 (35.0)	
**Dead with other diseases**	1 (4.0)	1 (10.0)	3 (7.5)	2 (5.0)	

*pCR* pathological complete response, *ESCC* esophageal squamous cell carcinoma

^*^Statistically significant

### The survival rate of patients with non-pCR after NACRT

**Fig. 1:**
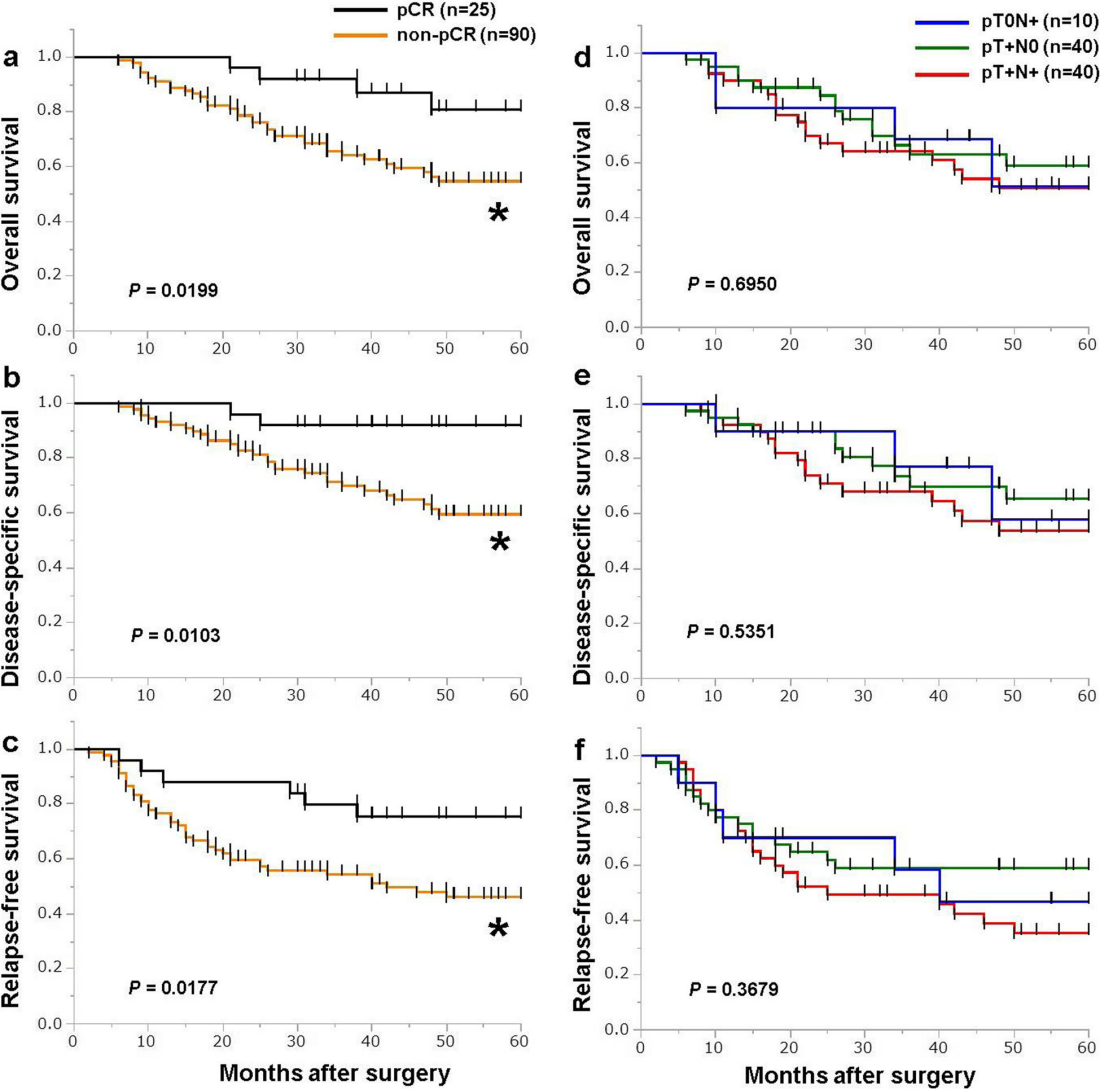
**a**–**c** Kaplan-Meier survival curves showing OS (**a**), DSS (**b**), and RFS (**c**) among the pCR group (black, *n*=25) and non-pCR group (orange, *n*=90). The log-rank test was used to compare the two groups. **d**–**f** Kaplan-Meier survival curves showing OS (**d**), DSS (**e**), and RFS (**f**) among pT0N+ group (blue, *n*=10), pT+N0 group (green, *n*=40), and pT+N+ group (red, *n*=40). The log-rank test was used to compare the three groups

Comparison of the survival rates between the pCR and non-pCR groups revealed that pCR patients had significantly better OS, DSS, and RFS than non-pCR patients (Fig. 1a–c). There were no significant differences in survival between pT0N+, pT+N0, and pT+N+ subgroups, though survival among the pT+N+ group was the poorest (Fig. 1d–f).

### Relationship between the SUV_max_ reduction rate in the primary tumor and survival in the pT+N+ group

**Fig. 2:**
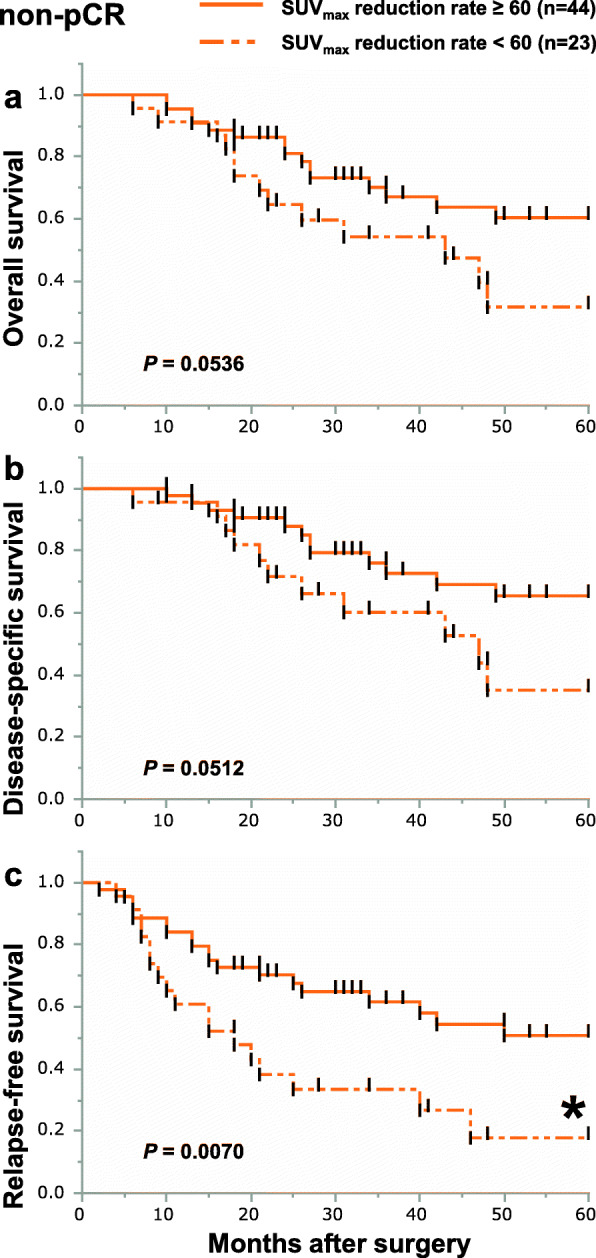
Kaplan-Meier survival curves showing OS (**a**), DSS (**b**), and RFS (**c**) according to the SUV_max_ reduction rate in the non-pCR group. Patients with SUV_max_ reduction rates of 60% or more in the primary tumor are indicated by solid lines (*n*=44); those with SUV_max_ reduction rates of less than 60% in the primary tumor are indicated by dotted-dashed lines (*n*=23)

**Fig. 3:**
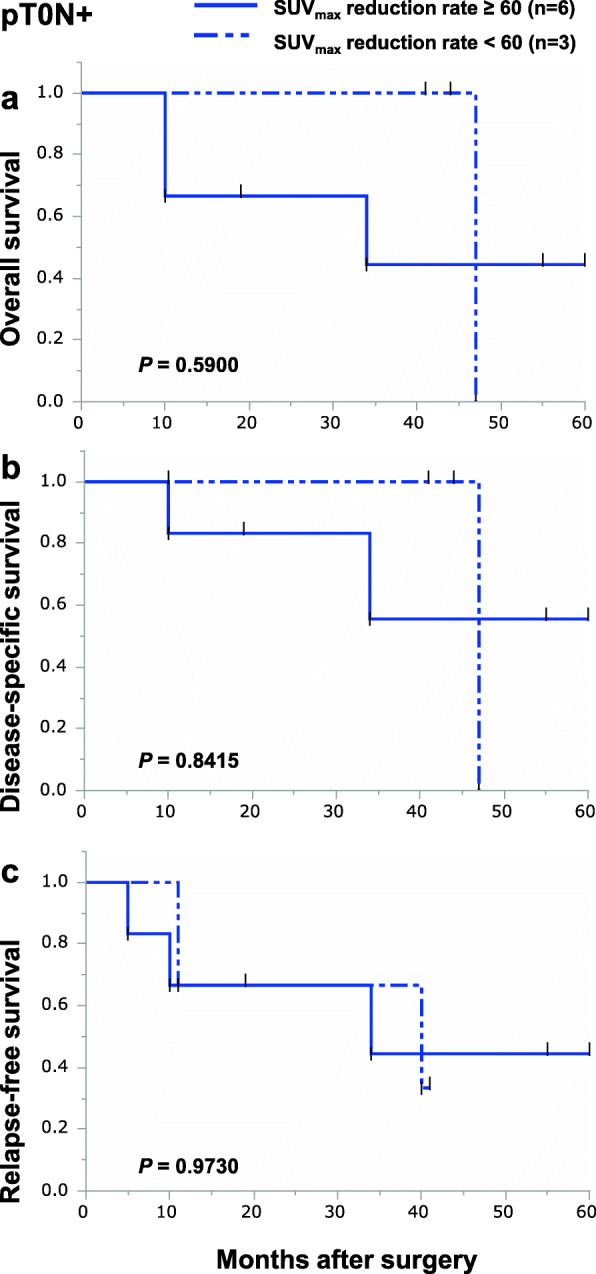
Kaplan-Meier survival curves showing OS (**a**), DSS (**b**), and RFS (**c**) according to the SUV_max_ reduction rate in the pT0N+ group. Patients with SUV_max_ reduction rates of 60% or more in the primary tumor are indicated by solid lines (*n*=6); those with SUV_max_ reduction rates of less than 60% in the primary tumor are indicated by dotted-dashed lines (*n*=3)

**Fig. 4:**
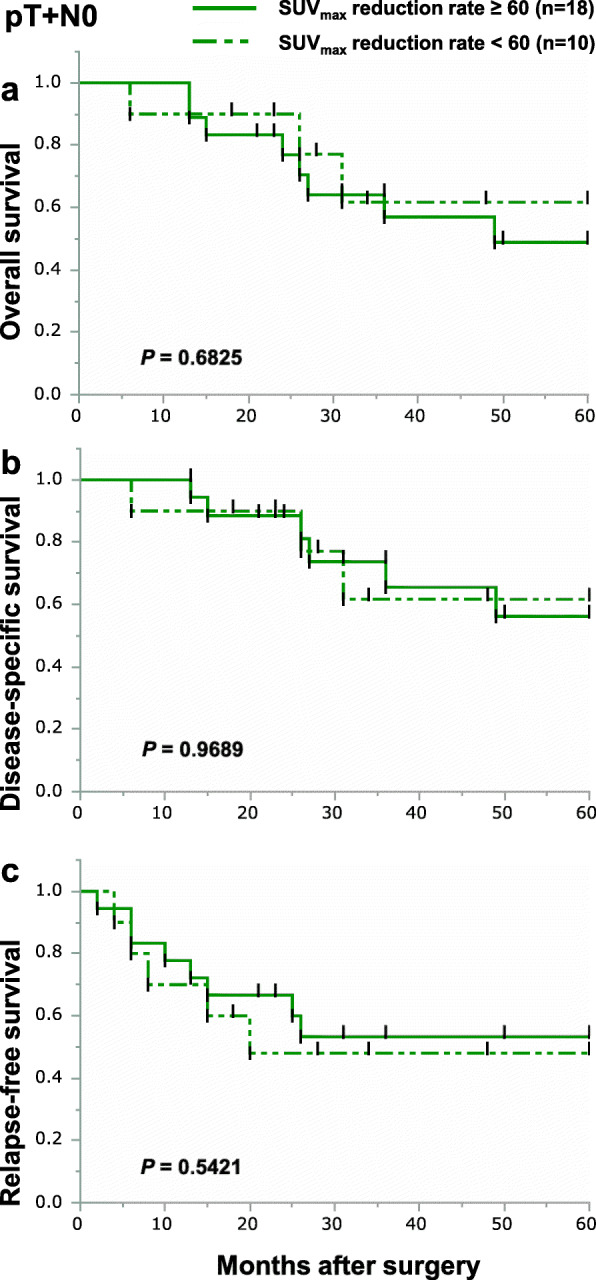
Kaplan-Meier survival curves showing OS (**a**), DSS (**b**), and RFS (**c**) according to the SUV_max_ reduction rate in the pT+N0 group. Patients with SUV_max_ reduction rates of 60% or more in the primary tumor are indicated by solid lines (*n*=18); those with SUV_max_ reduction rates of less than 60% in the primary tumor are indicated by dotted-dashed lines (*n*=10)

**Fig. 5:**
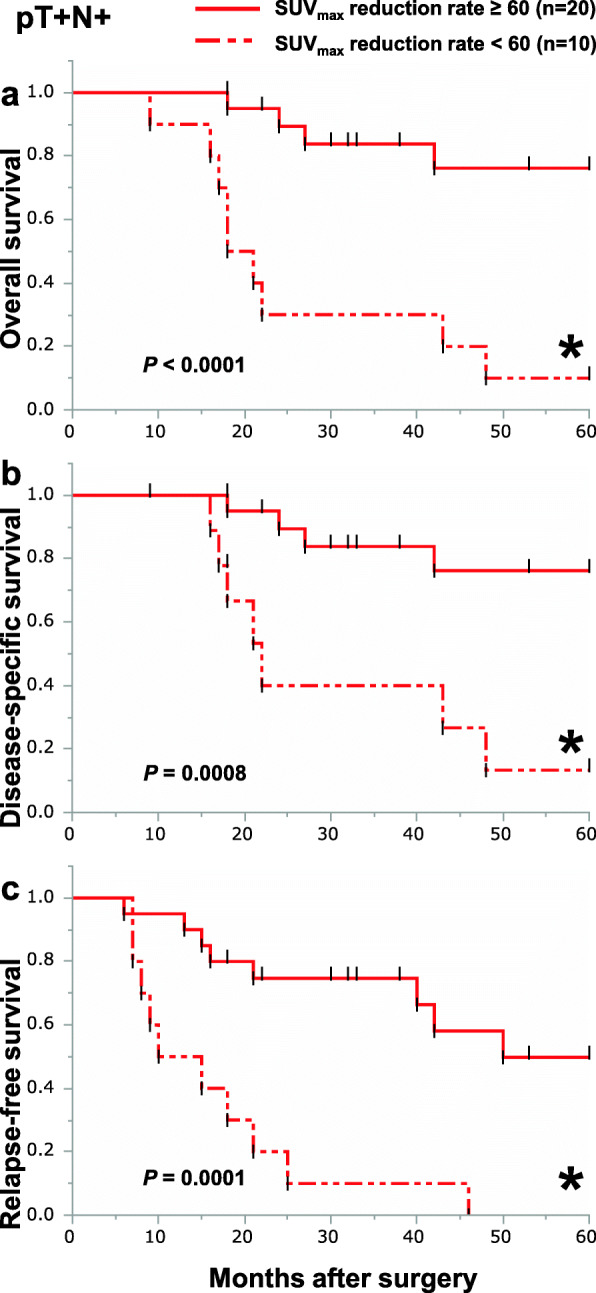
Kaplan-Meier survival curves showing OS (**a**), DSS (**b**), and RFS (**c**) according to the SUV_max_ reduction rate in the pT+N+ group. Patients with SUV_max_ reduction rates of 60% or more in the primary tumor are indicated by solid lines (*n*=20); SUV_max_ reduction rates of less than 60% in primary tumor are indicated by dotted-dashed lines (*n*=10)

We focused on the SUV_max_ reduction rate on FDG-PET measured in the primary tumors of 88 patients from whom SUV_max_ data were collected before and after NACRT. The median value of baseline SUV_max_ and post-NACRT SUV_max_ was 14.8 (2.9–42.0) and 3.2 (2.0–14.6), respectively. The interval between NACRT finished and FDG-PET taken was 20 (6–46) days. Based on the receiver operating characteristic (ROC) curve, the optimal cutoff value for the SUV_max_ reduction rate in the primary lesion for OS, DSS, and RFS was 60%. We divided non-pCR group by the optimal cutoff value for the SUV_max_ reduction rate. The clinicopathological characteristics of the two groups are shown in Table 2. We therefore performed Kaplan-Meier curve analyses about the two groups. Among all non-pCR patients, the 5-year OS and DSS were better in patients with a SUV_max_ reduction rate of 60% or more than in those with a SUV_max_ reduction rate of less than 60%, though the differences were not statistically significant (Fig. 2a, b). However, 5-year RFS for patients with SUV_max_ reduction rates of 60% or more was significantly better than that for patients with SUV_max_ reduction rates less than 60% (Fig. 2c). We next divided the non-pCR patients into the three subgroups and analyzed their prognosis using the optimal cutoff value for the SUV_max_ reduction rate. Among non-pCR patients, 9 (13.4%), 28 (41.8%), and 30 (44.8%) patients were categorized as pT0N+, pT+N0, or pT+N+, respectively. In the pT0N+ subgroup, there were no differences in OS, DSS, and RFS between patients with SUV_max_ reduction rates of more than 60% and less than 60% (Fig. 3a–c). In the pT+N0 subgroup, there were also no differences in OS, DSS, or RFS between patients with SUV_max_ reduction rates greater than or less than 60% (Fig. 4a–c). By contrast, in the pT+N+ subgroup, 5-year OS, DSS, and RFS among patients with SUV_max_ reductions rates of 60% or more were significantly better than among patients with SUV_max_ reduction rates less than 60% (Fig. 5a–c).

**Table 2:** Clinicopathological characteristics between two non-pCR group (SUVmax reduction rate (<60% or ≥60%)

Characteristic, ***n*** (%)	SUV_**max**_ reduction rate		***P***
	< 60%	≥ 60%	
	***n***=23	***n***=44	
**The interval days between NACRT finished and FDG-PET taken**	20 (10–46)	20 (6–32)	*0.5705*
**Median age (range)**	65 (47–75)	63.5 (41–75)	*0.5083*
**Gender**			0.7546
**Female**	3 (13.0)	7 (15.9)	
**Male**	20 (87.0)	37 (84.1)	
**Tumor location**			0.8080
**Upper**	5 (21.7)	10 (22.7)	
**Middle**	10 (43.5)	22 (50.0)	
**Lower**	8 (34.8)	12 (27.3)	
**Tumor differentiation**			0.7346
**Not poorly**	21 (91.3)	39 (88.6)	
**Poorly**	2 (8.7)	5 (11.4)	
**Depth of invasion (cT)**			0.7813
**T1-2**	2 (8.7)	3 (6.8)	
**T3-4**	21 (91.3)	41 (93.2)	
**Lymph node metastasis (cN)**			0.8980
**N1**	15 (65.2)	28 (63.6)	
**N2-3**	8 (34.8)	16 (36.4)	
**Distant metastasis (cM)**			0.0804
**M0**	19 (82.6)	42 (95.5)	
**M1 ( Lymph node)**	4 (17.4)	2 (4.6)	
**Clinical stage(cStage)**			0.0615
**I**	2 (8.7)	0 (0)	
**II**	0 (0)	3 (6.8)	
**III**	17 (7.39)	38 (86.4)	
**IVA**	0 (0)	1 (2.3)	
**IVB**	4 (17.4)	2 (4.6)	
**Recurrence**			0.0445^a^
**Presence**	15 (65.2)	17 (38.6)	
**Absence**	8 (34.8)	27 (61.4)	
**Prognosis**			0.1752
**Alive**	10 (43.5)	31 (70.5)	
**Dead with ESCC**	11 (47.8)	10 (22.7)	
**Dead with other diseases**	2 (8.7)	3 (6.8)	

^*^Statistically significant

### Prognostic analysis of OS, DSS, and RFS

We performed univariate and multivariate survival analyses with the pT+N+ group (Table 3). Univariate analysis revealed that age (≥65 or <65) and the SUV_max_ reduction rate in the primary tumor (<60% or ≥60%) were significant prognostic factors affecting 5-year OS and DSS in the pT+N+ subgroup. Moreover, univariate analysis of 5-year RFS in the pT+N+ group revealed that pathological depth of invasion (pT3-4 or pT1-2), pathological lymph node status (pN2-3 or pN1), and the SUV_max_ reduction rate in the primary tumor (<60% or ≥60%) were significant prognostic factors. Multivariate survival analysis of 5-year OS, DSS, and RFS in the pT+N+ group showed the SUV_max_ reduction rate was an independent prognostic factor. It thus appears that in pT+N+ ESCC patients, the SUV_max_ reduction rate is clinically useful for predicting 5-year OS, DSS, and RFS.

**Table 3:** Univariate and multivariate analyses of survival (Cox’s proportional hazards regression models)

Factor	Univariate	Multivariate	
	***P***-value	HR (95% CI)	***P***-value
**OS**			
**Age ( ≥65/<65)**	**0.0242**^a^	4.1216 (1.1369-14.9423)	**0.0311**^a^
**Gender (male/female)**	0.9989		
**Location (middle, lower/upper)**	0.1605		
**Differentiation (not poorly/poorly)**	0.6711		
**cT (T3-4/T1-2)**	0.9991		
**cN (N2-3/N1)**	0.7055		
**cM (M1 (lymph node)/M0)**	0.4804		
**pT (T3-4/T1-2)**	0.2130		
**pN (N2-3/N1)**	0.0965		
**SUV**_**max**_ **reduction rate in the primary lesion (<60%/≥60%)**	**0.0007**^a^	8.3800 (2.3893–29.3915)	**0.0009**^a^
**DSS**			
**Age ( ≥65/<65)**	**0.0246**^a^	5.2678 (1.2729–21.8008)	**0.0219**^**a**^
**Gender (male/female)**	0.9990		
**Location (middle, lower/upper)**	0.2356		
**Differentiation (not poorly/poorly)**	0.9992		
**cT (T3-4/T1-2)**	0.9992		
**cN (N2-3/N1)**	0.3436		
**cM (M1 (lymph node)/M0)**	0.3277		
**pT (T3-4/T1-2)**	0.4104		
**pN (N2-3/N1)**	0.0969		
**SUV**_**max**_ **reduction rate in the primary lesion (<60%/≥60%)**	**0.0034**^a^	7.6048 (1.9819–29.1798)	**0.0031**^a^
**RFS**			
**Age (≥65/<65)**	0.1947		
**Gender (male/female)**	0.2876		
**Location (middle, lower/upper)**	0.0759		
**Differentiation (not poorly/poorly)**	0.2910		
**cT (T3-4/T1-2)**	0.9203		
**cN (N2-3/N1)**	0.4032		
**cM (M1 (lymph node)/M0)**	0.7506		
**pT (T3-4/T1-2)**	**0.0312**^a^	2.5857 (0.8313–8.0423)	0.1008
**pN (N2-3/N1)**	**0.0330**^a^	0.6968 (0.1794–2.7072)	0.6019
**SUV**_**max**_ **reduction rate in the primary lesion (<60%/≥60%)**	**0.0006**^a^	6.2962 (1.4981–26.4625)	**0.0120**^a^

*OS* overall survival, *DSS* disease-specific survival, *RFS* relapse-free survival, *HR* hazard ratio, *CI* confidence interval

^*^Statistically significant

## Discussion

In the present study, we demonstrated that there was no significant survival difference among patients with ESCC categorized as pT0N+, pT+N0, or pT+N+ after treatment consisting of NACRT plus esophagectomy. Focusing on the SUV_max_ reduction rate on FDG-PET in the primary tumor, we observed that patients with a SUV_max_ reduction rate of less than 60% in the primary tumor after NACRT and categorized as pT+N+ after esophagectomy had the shortest 5-year OS, DSS, and RFS.

The development of multimodal therapies, such as chemotherapy, radiotherapy, and surgery, has improved the prognosis of esophageal cancer patients. The Chemoradiotherapy for Oesophageal cancer followed by Surgery Study (CROSS) trial found that NACRT plus surgery improved the survival rate among ESCC patients more than among esophageal adenocarcinoma patients [14]. After CROSS trial, NACRT followed by surgery was widely accepted for treatment of esophageal cancer, especially ESCC. Many studies have consistently shown that pCR after NACRT is a significant prognostic factor predictive of favorable long-term outcomes in ESCC [10, 15, 16]. Our finding that the 5-year OS rate in the pCR group was better than in non-pCR group is consistent with earlier reports.

While pCR patients obtained a benefit from NACRT followed by surgery, non-pCR patients did not. We therefore subdivided the non-pCR patients into pT0N+, pT+N0, and pT+N+ groups in effort to identify non-pCR patients who might benefit from NACRT followed by surgery. Several earlier reports have suggested that pathological N stage after NACRT is the most important prognostic factor in ESCC and that the number of pathological metastatic lymph nodes after NACRT was the strongest indicator of patient survival [17]. Patients without pathological lymph node metastasis after NACRT followed by surgery were associated with a good prognosis, even when the CRT effect on the primary tumor was poor [18]. We therefore expected that patients with pT0N+ and, especially, pT+N0 would have better prognoses than those with pT+N+; however, no difference was found in their prognoses. Hatogai et al. showed that the pN factor based on the number of metastatic lymph nodes in the current staging system can predict a poor outcome among ESCC patients who have received NACT followed by surgery, but the pN factor was not an independent prognostic factor [19]. They showed that the pathological tumor regression grade for main tumor and metastatic tumors in lymph nodes is predictive of prognosis in esophageal cancer patients who received NACT followed by surgery [19, 20]. In the present study, the survival curve for the pT+N+ group was lowest among the three non-pCR groups, but there were no significant differences. For that reason, we focused on the SUV_max_ reduction rate on FDG-PET.

FDG-PET is useful for the diagnosis of metastasis before treatment and recurrence after treatment, and it can also be used to evaluate the response to neoadjuvant therapy [21, 22]. An earlier study indicated that the SUV_max_ reduction rate in the primary tumor may be a more significant predictor of CR after NACRT than the change in metabolic tumor volume (MTV) or total lesion glycolysis (TLG), two other parameters measured through FDG-PET examination [23]. The optimal cutoff for the SUV_max_ reduction in the primary tumor was 72% for pCR prediction after NACRT [23]. Another recent report found that the SUV_max_ reduction rate in the primary tumor is a valuable predictor of the pathological response to NACRT and survival [11]. That report showed that, using a cut off of 75%, the SUV_max_ reduction rate in the primary tumor can predict pCR and that by using a cut off of 70%, the SUV_max_ reduction rate in the primary tumor was an independent prognostic factor associated with better DSS [11]. Those findings suggest that the degree of tumor metabolic response determined by preoperative FDG-PET is an important preoperative prognostic indicator for patients undergoing NACRT; moreover, the magnitude of the difference between the metabolic activities in primary tumors before and after NACRT is a useful guide for establishing postoperative therapy and postoperative surveillance programs. However, the cutoff values differ widely among studies, and the optimal cutoff value has not been determined. In the present study, ROC curve (data not shown) analysis showed that a SUV_max_ reduction rate of 60% in the primary tumor was the optimal cutoff for predicting survival in non-pCR ESCC patients after NACRT. We suggest this cutoff value was lower than in previous reports because this analysis was limited to non-pCR patients among ESCC patients who underwent surgery after NACRT.

The results of this study show that patients with a SUV_max_ reduction rate of less than 60% in the primary tumor after NACRT and categorized as pT+N+ after esophagectomy have an especially poor prognosis. We suggest that these patients should receive more powerful postoperative adjuvant therapy that differs from the preoperative chemotherapy regimens (e.g., immune checkpoint inhibitors). In addition, we also suggest that patients with a SUV_max_ reduction rate of less than 60% in the primary tumor avoid surgery at that time and opt for stronger chemotherapy if pT+N+ after NACRT is clearly expected based on preoperative imaging examinations. It would then be advisable to consider surgery after determining the effectiveness of added chemotherapy.

We are aware that some limitations were inherent in this study. The retrospective nature of this study may have introduced selection bias. Because this study included only a small number of cases at a single institution, its impact is limited. In the future, a prospective research design in collaboration with other facilities will be required.

## Conclusions

The SUV_max_ reduction rate after NACRT is a highly useful predictor of prognosis in non-CR patients, especially patients who are pT+N+ after NACRT and esophagectomy. Clinical non-CR patients with SUV_max_ reduction rates less than 60% on FDG-PET should be treated with additional adjuvant therapy before or after esophagectomy.

## Data Availability

The datasets used and/or analyzed during the current study are available from the corresponding author on reasonable request.

## Abbreviations

pCR: Pathological complete response
NACRT: Neoadjuvant chemoradiotherapy
NACT: Neoadjuvant chemotherapy
ESCC: Esophageal squamous cell carcinoma
OS: Overall survival
DSS: Disease-specific survival
RFS: Relapse-free survival
FDG-PET: ^18^F-fluorodeoxyglucose-positron emission tomography
SUV_max_: The maximum standardized uptake value
ECOG PS: Eastern Cooperative Oncology Group performance status
5-FU: 5-fluorouracil
CTCAE: Common Terminology Criteria for Adverse Events
CROSS: Chemoradiotherapy for Oesophageal cancer followed by Surgery Study
